# Feasibility and pilot study of passive heat therapy on cardiovascular performance and laboratory values in older adults

**DOI:** 10.1186/s40814-023-01314-1

**Published:** 2023-05-23

**Authors:** Brigid Flynn, Michelle Vitztum, Joshua Miller, Abigail Houchin, Jaromme Kim, Jianghua He, Paige Geiger

**Affiliations:** 1 Department of Anesthesiology, University of Kansas Medical Center, 3901 Rainbow Blvd., Kansas City, KS 66160 USA; 2 Department of Molecular and Integrative Physiology, University of Kansas Medical Center, 3901 Rainbow Blvd., Kansas City, KS 66160 USA; 3 University of Kansas Medical Center, 3901 Rainbow Blvd., Kansas City, KS 66160 USA; 4 Department of Biostatistics and Data Science, University of Kansas Medical Center, 3901 Rainbow Blvd., Kansas City, KS 66160 USA

**Keywords:** Heat therapy, Hot tub, Cardiovascular, Arterial, Distensibility, Endothelial, Hemodynamic

## Abstract

**Background:**

Chronic heat therapy may have beneficial effects on cardiovascular function. These effects may be more pronounced in older adults. We performed a pilot feasibility study of repeated heat therapy sessions in a hot tub (40.5 °C) in older adults while wearing a noninvasive hemodynamic monitor. As part of the protocol, the volunteers underwent cardiovascular performance testing pre- and post-intervention.

**Methods:**

Fifteen volunteers > 50 years old underwent 8–10 separate 45-min hot tub session over 14 days in this exploratory and mixed methods trial. The participants had maximal oxygen consumption (VO_2_ max) and other cardiovascular data measured via exercise treadmill testing prior to and after all hot tub sessions. The participants also wore noninvasive fingertip volume clamp monitors while immerged in hot water that calculated systemic vascular resistance, heart rate, blood pressure, and cardiac output in order to ascertain the feasibility and utility of this data. Other laboratory studies were obtained pre- and post-intervention. The protocol was determined feasible if the heat therapy and cardiovascular testing was completed by at least 90% (14/15 subjects). Feasibility of the noninvasive monitor was determined by the fidelity of the results. Secondary exploratory outcomes were analyzed for differences to identify if they are acceptable to include in an efficacy trial.

**Results:**

All participants completed the study protocol identifying the feasibility of the protocol. The noninvasive hemodynamic monitors successfully recorded cardiac output, systemic vascular resistance, heart rate, and blood pressure with fidelity based on the analysis of recordings. In the secondary analyses, we found no difference in the pre- to post-intervention measurement of VO_2_ max but did find increased exercise duration following hot tub therapy compared with prior to the therapy (571 s versus 551 s).

**Conclusions:**

The current pilot study protocol is feasible for the purpose of analyzing the effects of heat therapy and cardiovascular performance in older adults while wearing a noninvasive hemodynamic monitor and undergoing treadmill stress testing. Secondary analyses found increased exercise tolerance but no differences in VO_2_ max following heat sessions.

## Key messages regarding feasibility

• It is unknown if older adults, who may most benefit from the potential effects of heat therapy on cardiovascular fitness, can tolerate sequential hot tub immersions and perform cardiovascular fitness testing on a treadmill. It is also unknown if a noninvasive finger cuff can record hemodynamic variables while volunteers are immerged in hot water.
• Older adults can tolerate hot water immersion for eight to ten, 45-min sessions with only minor hemodynamic and symptomatic changes. A noninvasive finger cuff can ascertain key hemodynamic data while volunteers are immerged in hot water.
• Older adults are able to complete hot tub sessions. Older adults can perform the associated exercise testing in order to test the cardiovascular changes secondary to heat therapy. Finally, a noninvasive finger cuff can provide hemodynamic data in order to ascertain the effects of hot water immersion on the human body.

## Background

Millions of people worldwide suffer from cardiovascular disease. Cardiovascular disease is the leading cause of death for both men and women in the USA [1]. Compromised endothelial function has been implicated in the development and progression of cardiovascular disease due to arterial stiffness, inflammation, and lack of vascular relaxation. The arterial endothelium is recognized as a smart barrier and key regulator of blood flow in microvascular and macrovascular beds [2].

Previous work has demonstrated that heat exposure can improve arterial endothelial function [3–7]. In sedentary adults, heat therapy via water immersion has been shown to have widespread beneficial effects on vascular dilatation and a reduction in arterial stiffness [8]. Furthermore, acute hot water immersion has been shown to provide similar or greater beneficial vascular changes when compared with acute exercise [6, 7]. However, it is unknown if older adults—a group that may most benefit from improved vascular distensibility—can tolerate sequenced hot tub immersions along with cardiovascular fitness measurements performed with treadmill exercise testing. It is also unknown if a finger cuff noninvasive hemodynamic monitor will be provide fidelity in measurements while volunteers are immerged in hot water.

We sought to determine the feasibility of a protocol involving cardiovascular fitness testing prior to and after sequential heat immersion therapy in hot tubs in older adults. Feasibility was deemed adequate if 90% of participants completed all sessions of exercise testing and hot tub sessions without stopping due to hemodynamic or environmental intolerability. We also tested the feasibility of using of a noninvasive hemodynamic monitor while volunteers were immerged in water based on fidelity of the measurements. The secondary aim was to perform a pilot analyze on the impact of heart therapy on cardiovascular fitness, by analyzing results of the fitness measurements prior to after heat immersion therapy sessions.

## Methods

The aim of this exploratory, pilot study was to ascertain feasibility of a protocol in which older adults undergo 8–10 sequential 45-min heat therapy sessions in a hot tub within a 2-week period, along with exercise and laboratory tests of cardiovascular fitness. The decision to complete 8 versus 9 versus 10 hot tub sessions was dictated by volunteer availability during the 2-week study period. Feasibility of obtaining high fidelity data with a noninvasive finger cuff hemodynamic monitor was also tested while participants were immerged in hot water. Fidelity of these measured was assessed by waveform and numerical values that were monitored in real time by a study proctor and post-intervention by other members of the research team. All hot tub sessions and testing were performed at a translational research center affiliated with a university, tertiary care center. The secondary aim was to analyze outcome data from the exercise testing to identify if differences were noted from the pre-intervention testing to the post-intervention testing.

After institutional review board approval, study participants were recruited by advertisement fliers posted on public bulletin boards at a tertiary care hospital and medical school, the university Research Study Opportunities webpage, and word of mouth. Volunteers were required to be 50 years or older and in good physical condition without major cardiopulmonary, renal, or hepatic comorbidities based on self-reporting.

As a pilot study, the number of participants necessary to assess feasibility could not be ascertained from previous literature involving older adults. Thus, the number needed was estimated based similar literature in healthy, young adults and also the researchers’ personal experiences in the heat therapy study space [5, 6]. Approximately 15 participants was the initial goal. Once this was achieved and due to time and resource constraints, recruitment was stopped. In total, 15 participants volunteered, were pre-screened to ensure inclusion and exclusion criteria were met, and ultimately entered into the study (Table 1). All participants provided oral and written informed consent prior to participation. Monetary compensation of $100 was offered upon completion of the study.

**Fig. 1:**
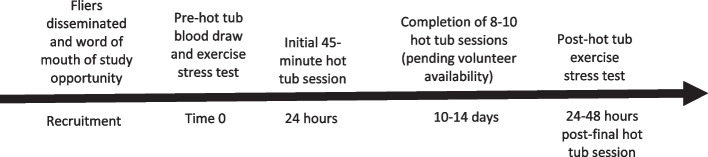
A timeline of the study

Prior to heat immersion therapy, all participants had baseline blood samples taken the day prior to the initial hot tub session and included complete metabolic panel, lipid panel, and complete blood counts (Table 2). If the participant was of childbearing potential, a urine pregnancy test would have been required to ensure the participant was not pregnant. However, this was not necessary in this population. Participants also underwent Bruce stress protocol treadmill exercise testing [9] the day prior to the first hot tub session to ascertain the primary outcome of changes in VO_2_ max, along with other secondary outcome measurements (Table 2). Measurement of VO_2_ max and obtainment of blood samples were performed with the Bruce treadmill stress test protocol at two times: prior to the first heat therapy session and 24–48 h following the last emersion from the hot tub. Figure 1 illustrates the timeline of the study.

All participants completed the required 8–10 with six participants completing 10 sessions, five participants completing 8 sessions, and four participants completing 9 sessions. The participants underwent shoulder-high, heated (40.5 °C) water immersions of 45 min each in hot tubs within a 14-day period. A 2-week period was utilized in order to achieve chronic changes in endovascular health instead of immediate, acute changes. Dry body weight was measured prior to and after each hot water therapy. If body weight loss following therapy was > 1%, the participant was asked to drink additional fluids to make up the difference. Participants had vital signs assessed prior to each heat immersion session. If pre-therapy systolic blood pressure was < 75 mmHg or > 180 mmHg and/or diastolic blood pressure < 40 mmHg or > 90 mmHg, participation would not be allowed.

Sterile rectal thermistor probes inserted approximately 2.54 cm past the anal sphincter monitored the participant’s temperature. The participant was removed from the hot tub if rectal temperature probe recorded a temperature of 38.5 °C or higher and asked to sit on the side of the hot tub until < 38.0 °C at which time they were asked to return into the hot tub. Participants were able to drink water ad libitum while in the hot tub. Rectal temperatures were monitored for 10 additional minutes following emersion from the hot tub or until < 38.0 °C.

Following all hot tub sessions, a second treadmill exercise test was performed 24–48 h following emersion from heat therapy. This waiting period was adhered to in order to obviate the transient enhancements of cardiac output and systemic vascular resistance due to water immersion that may alter VO_2_ max assessments [6, 7]. This ensured that any changes in VO_2_ max were not due to acute benefits of heat therapy, but more chronic changes. This waiting period also allowed for muscle rest prior to the exercise testing in order to ensure changes in VO_2_ max were not due to fatigue.

While in the hot tub, participants wore a noninvasive fingertip blood pressure hemodynamic monitor (Clearsight, Edwards, Lifesciences, Irvine, CA) [10] that continually recorded and calculated cardiac output, systemic vascular resistance (SVR), stroke volume, heart rate, and blood pressure. If blood pressure or heart rate deviated significantly from baseline, the participant was assessed for neurologic changes. If the deviation was 50% or more, the participant was asked to sit on the side of the hot tub until vital signs returned to baseline. If post-hot tub blood pressure varied by > 20% lower or > 30% higher than baseline values, the subject was monitored until return to < 20% deviation from baseline prior to discharge from the facility. If blood pressure values did not return to baseline after 10 min, the plan was to discuss with an on-site medical provider. The decision for 45 min per session was incorporated in order to achieve steady state of these changes in each participant.

Descriptive statistics (mean and standard deviation) were used to summarize the pre and post values as well as the changes (post–pre) for each outcome. For each data point per volunteer, changes in average values from the first and last exercise stress test sessions (weight, maximum heart rate, exercise duration, respiratory exchange ratio, laboratory values) or the first and last hot tub sessions while immersed (cardiac output, systemic vascular resistance, heart rate, systolic, diastolic and mean blood pressures) were used for analysis. Wilcoxon signed-rank test was used to test whether the pre-post change for each variable is statistically significant or not (*p* < 0.05). We also used line plots to show the raw data and the changing patterns. All analyses were conducted using software R version 40.3

## Results

All 15 participants who entered the study completed the study protocol identifying the feasibility of the protocol. Table 1 demonstrates the baseline characteristics of the subjects. Only one subject experienced an adverse event consisting of dizziness immediately after exiting the hot tub. However, the subject recovered during the 10-min cooldown period. Symptoms did not persist at future visits. No subjects had to be removed from the hot tub during treatment. No subjects had to stop intervention.

**Table 1:** Baseline characteristics of the participants (*n* = 15)

	*n* = 15
Age (years)	58.6 (50–76)
Female	9 (60%)
Race (6 did not answer)	
White	7 (88%)
Asian	1 (12%)
Weight (kg)	80.9 (48–120)
Hypertension	3 (21%)
Diabetes	2 (14%)
Asthma	2 (14%)

Continuous variables given as mean (minimum, maximum)

In addition, feasibility was also identified with the high fidelity hemodynamic measurements from the noninvasive finger cuff provided. Acceptable continuous data was obtained while the volunteers were immersed in the hot tub. Fidelity of waveform and numerical data was monitored in real-time by a trained study proctor at all times to ensure accuracy of data. Additionally, the volunteers did not have difficulty with the necessary placement of the finger cuff hand outside of the hot tub in order to remain dry. Since the finger cuff was placed on the volunteer after immersing in water and the volunteers remained seated during the experience, there were no issues with water splashing or electrical risk encountered.

**Fig. 2:**
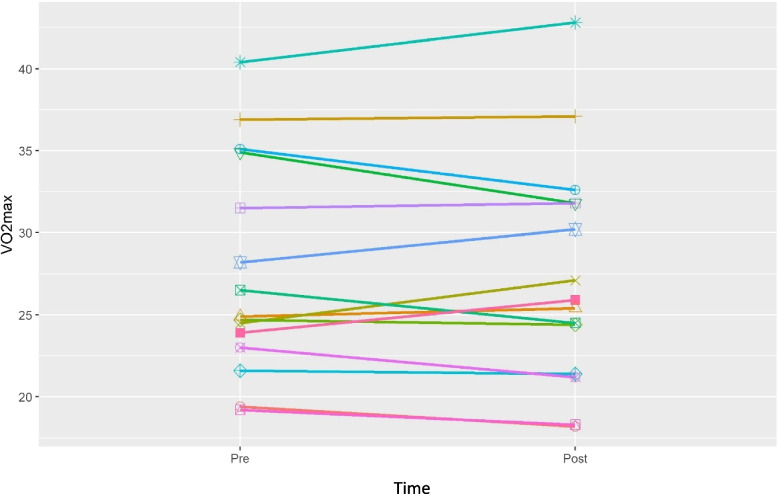
Line graph representation of changes in VO_2_ max from pre- to post-intervention in the 15 subjects. There was no statistical difference in VO_2_ max from the first exercise testing prior to the hot tub sessions to the last exercise testing following the hot tub sessions (− 0.20 (95% CI − 1.3, 1.1))

**Fig. 3:**
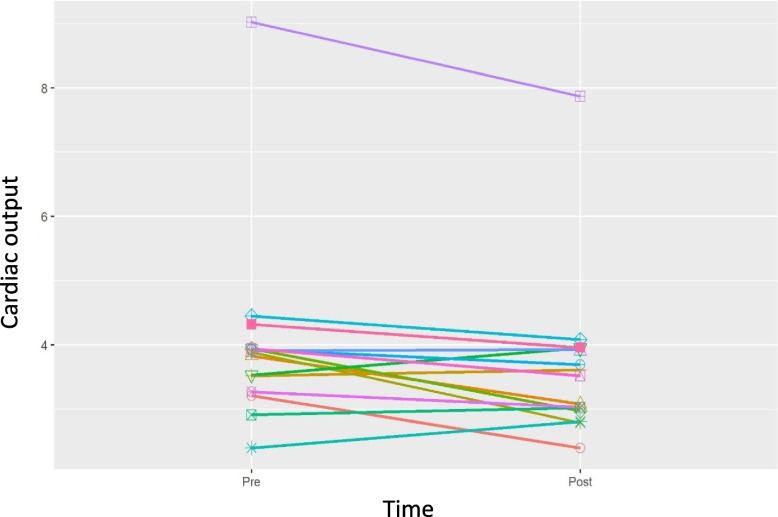
Line graph of cardiac output measurements in the 15 subjects from the first hot tub session to the final hot tub session. Cardiac output was measured by a noninvasive, fingertip blood pressure cuff utilizing volume clamp and pulse contour technology

The secondary outcomes of the pilot study are displayed in Table 2. Pre- and post-hot tub therapy cardiovascular fitness results parameters were reported as average per participant over the total hot tub sessions. There was no change in VO_2_ max (− 0.20 (95% CI − 1.3, 1.1)) (Fig. 2). Cardiac output had a trend of significance (− 0.36 (95% CI − 0.67, − 0.005)) (Fig. 3); however, much of this trend can be attributed to one participant. Exercise duration was longer post-hot tub therapy (571 s versus 551 s (22 (95% CI (2.5, 36)). Other outcomes of cardiovascular fitness were unchanged from per- to post-intervention. Outcomes of laboratory data were also unchanged following intervention.

**Table 2:** Changes in average values for each volunteer from the first and last exercise stress test sessions (weight, maximum heart rate, exercise duration, respiratory exchange ratio, laboratory values) or the first and last hot tub sessions to (cardiac output, systemic vascular resistance, heart rate, systolic, diastolic and mean blood pressures) (*n* = 15)

	Pre	Post	Change	*p*-value	Median (95% confidence interval)
Exercise stress testing data					
VO_2_ max (ml/kg/minute)	27.7 (6.6) (19.2, 40.4)	27.5 (6.9) (18.2, 42.8)	− 0.1 (1.8) (− 3.1, 2.6)	0.842	− 0.20 (− 1.3, 1.1)
Weight (kg)	79 (23.5) (48.1, 120)	80 (23.7) (48.1, 120.7)	0.7 (1.6) (− 1.1, 5.4)	0.09	0.40 (− 0.4, 0.4)
Maximum heart rate (beats per minute)	165 (12) (146, 188)	163 (14) (138, 192)	− 2 (6) (− 15, 5)	0.325	0.00 (− 1, 1)
Exercise duration (seconds)	551.7 (122) (278, 707)	571.4 (112.2) (355, 741)	19.7 (30.2) (− 39, 77)	0.026	22.00 (2.5, 36)
RER max	1.2 (0.1) (1.1, 1.4)	1.2 (0.1) (1.1, 1.3)	− 0.02 (0.1) (− 0.1, 0.1)	0.147	− 0.02 (− 0.035, 0.015)
Hot tub session data					
Cardiac output (L/minute/m^2^)	4 (1.5) (2.4, 9)	3.7 (1.2) (2.4, 7.9)	− 0.3 (0.5) (− 1.2, 0.4)	0.044	− 0.36 (− 0.67, − 0.005)
Systemic vascular resistance (dynes/seconds/cm^−5^)	967 (371) (679, 1960)	975.9 (269) (617, 1559)	9.0 (269) (− 612, 378)	0.583	41.55 (− 136.9, 161.7)
Heart rate (beats per minute)	89 (12) (62, 106)	88 (10) (66, 103)	− 0.6 (6) (− 10, 12)	0.561	− 2.23 (− 4.3, 2.9)
Systolic blood pressure (mmHg)	106 (13) (86, 131)	103 (12) (85, 121)	− 3 (11) (− 22, 21)	0.182	− 2.30 (− 8.17, 2.64)
Diastolic blood pressure (mmHg)	65 (11) (48, 99)	66 (10) (50, 97)	0.9 (5) (− 6, 9)	0.639	1.10 (− 2, 3.55)
Mean arterial pressure (mmHg)	79 (8) (63, 91)	79 (7) (63, 90)	− 0.6 (8) (− 12, 15)	0.561	− 1.60 (− 4.95, 3.54)
Laboratory values					
Total cholesterol (mmol/L)	192 (29) (148, 253)	192 (27) (139, 229)	− 0.1 (15) (− 28, 22)	1	4.00 (− 9, 9)
HDL cholesterol (mmol/L)	60 (11) (41, 79)	61 (13) (37, 84)	0.7 (5) (− 7, 10)	0.588	1.00 (− 2, 3.5)
LDL cholesterol (mmol/L)	105 (24) (72, 150)	105 (26) (59, 147)	− 0.5 (11) (− 17, 16)	0.925	− 3.00 (− 8.5, 6)
Creatinine (mg/dL)	0.9 (0.2) (0.7, 1.2)	0.9 (0.1) (0.7, 1.1)	− 0.01 (0.1) (− 0.1, 0.1)	0.552	0.00 (− 0.07, 0.04)
Albumin (g/dL)	4.3 (0.3) (3.9, 4.7)	4.3 (0.3) (3.8, 4.7)	0.02 (0.2) (− 0.2, 0.4)	0.918	0.00 (− 0.15, 0.15)
AST (units/L)	21.2 (7.5) (11, 42)	19.2 (5.3) (12, 29)	− 2.5 (5.4) (− 15, 2)	0.21	− 0.50 (− 1.5, 1.5)
ALT (units/L)	21.5 (10.2) (10, 52)	19.53 (6.5) (11, 37)	− 2.1 (5.1) (− 15, 3)	0.222	− 1.00 (− 1.5, 1.5)
WBC (10^9^/L)	5.7 (1.3) (4.1, 9)	5.7 (1.0) (4, 7.5)	0.02 (1.1) (− 2.7, 1.5)	0.755	− 0.30 (− 0.55, 0.6)
Hemoglobin (g/dL)	13.7 (1.1) (11.8, 16.6)	13.7 (1.5) (9.7, 16)	0.03 (1.1) (− 3.3, 1.2)	0.345	0.30 (− 0.4, 0.4)
Platelet (10^9^/L)	2681 (77) (183, 463)	278 (79) (162, 428)	10 (27) (− 35, 58)	0.191	8.00 (− 4.5, 29)

Wilcoxon signed-rank test used for data analysis. Data presented as mean (standard deviation) (maximum, minimum) or as median (95% confidence interval). *RER* Respiratory exchange ratio, *HDL* High-density lipoprotein, *LDL* Low-density lipoprotein, *AST* Aspartate aminotransferase, *ALT* Alanine transaminase, *WBC* White blood cells

## Discussion

In this study, we tested the feasibility of consecutive heat therapy sessions and exercise tests of cardiovascular fitness in older adults. We also tested the feasibility wearing a noninvasive finger cuff hemodynamic monitor while immersed in the hot tub. The study protocol of 8–10 sequential 45-min heat therapy sessions in a hot tub within a 2-week period, along with pre- and post-intervention cardiopulmonary exercise testing on a treadmill, was tolerated by all 15 participants. Feasibility was also demonstrated in the fidelity of data from the hemodynamic monitor.

These findings demonstrate that further efficacy studies could be performed with this protocol while maintaining tolerability in participants. This is important in that heat therapy may be beneficial for endothelial function and cardiopulmonary fitness. Older adults were chosen for this study as this demographic may realize the most benefit from beneficial effects of heat therapy on endovascular and arterial function. Additionally, older adults may be a group that would not tolerate consecutive hot tub therapy sessions of 45 min each, and, hence, this feasibility study was undertaken.

We found that all 15 participants were able to tolerate the hot tub sessions and also complete the required pre- and post-intervention treadmill exercise testing. The protocol utilized in this study consisted of 8–10 sessions over a 14-day period in hopes of developing “chronic” cardiovascular benefits of heat therapy instead of acute benefits as ascertained by previous studies [3, 4].

We also found that all subjects were able to be successfully monitored with a fingertip blood pressure cuff with reliable readings of heart rate, blood pressure, and calculations of SVR and cardiac output while in the hot tub for the entirety of each session. The technology utilized in this novel method of SVR and cardiac output calculation is performed through volume clamping technology in which equal pressures are dynamically provided on either side of the wall of the artery by clamping the artery to a certain constant volume [10]. Since this arterial distensibility can be affected by both water immersion and heat, it was important to identify if this is a viable hemodynamic monitor for hot tub studies [11].

In the secondary aim of the pilot study, we observed no difference in the main outcome of cardiovascular function and resultant exercise tolerance as measured by VO_2_ max (Table 2, Fig. 2). Cardiac output did have a signal toward significance; however, this may have been due to one outlying subject (Fig. 3). Additionally, the participants did have an increase in exercise duration of a mean of 20 s following the hot tub sessions. Other measures of cardiovascular fitness and laboratory data were unchanged by heat therapy intervention.

Understanding hemodynamic changes during water immersion and heat is valuable as additional measures of cardiopulmonary evaluation. It is known that heart rate typically decreases in neutral temperature water by 12–15% but increases significantly in warm water contributing to yet a further rise in cardiac output at high temperatures [12, 13]. Additionally, the increase in SVR is off-set by increasing temperature [14] and must be accounted for by strict hot tub temperature management, volunteer rectal temperature monitoring, and volunteer compliance. Due to these inter-related cardiovascular changes, the current protocol required participants to remain in each hot tub session for 45 min in order to establish steady state for these effects of hot water immersion.

Understanding the effects of heat therapy on cardiovascular health with treadmill exercise testing is also important as cardiovascular comorbidities are currently a leading cause of morbidity and mortality in many countries. Healthy, distensible arteries allow smoothing of blood pressure variations and ensure blood flow in one direction to all vital organ systems. As arteries stiffen and lose elasticity, pulsatility is dampened causing wider blood pressure fluctuations within the micro- and microvasculature placing vital organs such as the heart, brain, and kidneys at risk [15]. Endothelial dysfunction coincides with a decreased production of endogenous vasodilatory mediators, such as nitric oxide, worsening the nondistensibility, and lack of vasodilation. Ultimately, this lack of elasticity leads to failure of regulation of blood flow and vital, end-organ damage results. However, if heat therapy does not translate into clinical benefit, other modalities of improving arterial distensibility must be sought [16].

This study was undertaken due to previous work demonstrating that heat exposure can improve arterial endothelial function in both acute and chronic therapy studies [3–7]. In acute, one-time passive heat versus exercise studies, hot-water immersion increased major artery blood flow and shear rate equivalent to exercising at a moderate intensity [6, 7]. In more chronic studies, heat therapy via water immersion (4–5 times per week for 8 weeks) had beneficial effects on vascular dilatation and a reduction in arterial stiffness as measured by increased flow-mediated dilatation, improved arterial compliance, reduced aortic pulse wave velocity, decreased carotid intima media thickness, and decreased blood pressure [5]. However, it is not known what protocol is best and if older adults, who may benefit most, could tolerate sequential heat therapies and exercise test of cardiopulmonary function.

Other previous work in healthy, younger patients has used a protocol consisting of 8 weeks of heat therapy [16]. In other studies, in acute, one-time heat versus exercise studies, the beneficial effects were noted immediately after intervention. However, we chose to not obtain cardiovascular parameters immediately following heat therapy in order to avoid confounding factors, such as fatigue and dehydration, following a hot tub session, and hence, this could account for differences in our findings.

The present study identified feasibility in a protocol requiring that older adults, who theoretically would most benefit from the potential effects of heat therapy on cardiovascular fitness, are able to tolerate 8–10 hot tub sessions of 45 min each within a 14-day period. Additionally, older adults can perform the associated exercise testing in order to test the cardiovascular changes secondary to heat therapy. This study also identified feasibility in fidelity of hemodynamic data ascertained from a noninvasive finger cuff while participants are immerged in hot water. This particular finding lays the groundwork for future studies aiming to understand the effects of hot water immersion on the human cardiovascular system, including heart rate, blood pressure, systemic vascular resistance, and cardiac output.

## Data Availability

All data is available upon request.
